# Improving T-cell engager efficacy in glioblastoma with multi-antigen targeting and novel delivery approaches

**DOI:** 10.1007/s00401-026-03063-w

**Published:** 2026-08-03

**Authors:** Arushi Tiwari, Kristen D. Pawlowski, Irina V. Balyasnikova

**Affiliations:** 1 Department of Neurological Surgery, Northwestern Medicine Malnati Brain Tumor Institute of the Lurie Comprehensive Cancer Center, Feinberg School of Medicine, Northwestern University, 303 E Superior St., Chicago, IL 60611 USA; 2 Department of Neurology, University of Michigan, 1500 E Medical Center Dr., Ann Arbor, MI 48109 USA

## Abstract

Glioblastoma remains a challenging disease to approach with immunotherapy due to pronounced antigen heterogeneity, immunosuppressive tumor microenvironment, and barriers to effective molecule delivery within the central nervous system. T-cell engagers provide an off-the-shelf approach to redirect endogenous T cells toward tumor cells. However, bispecific formats are constrained by intra- and interpatient antigen heterogeneity, which can limit therapeutic efficacy. In this review, we examine trispecific T-cell engagers (TriTEs) as an emerging strategy to address this limitation by simultaneously targeting multiple tumor-associated antigens. We discuss principles guiding antigen selection in glioblastoma, summarize available preclinical evidence supporting multispecific engagement, and outline key design considerations, including molecular architecture, stability, half-life extension, and safety optimization. We further review delivery strategies, such as gene-encoded expression, cellular carriers, and blood–brain barrier modulation, that may improve tumor access and the durability of TriTEs. Together, these considerations position TriTEs as a modular immunotherapy platform relevant to glioblastoma and other heterogeneous solid tumors.

## Introduction

### Glioblastoma

Glioblastoma (GBM) is the most aggressive primary brain tumor in adults, with an annual incidence of 3.6 per 100,000 people [33, 98]. The standard of care consists of maximal surgical resection followed by concurrent fractionated radiotherapy and oral temozolomide (TMZ) chemotherapy [120], and then maintenance TMZ with optional tumor-treating fields adjuvant therapy to improve overall survival [17]. Despite first-line treatment, GBM has a near-universal recurrence rate, a median survival of 15–17 months, which extends to 20.9 months with tumor-treating fields, and a 5-year survival rate of approximately 3% [100, 119, 121]. Without an established standard of care for recurrent disease, management options are limited to repeat surgical resection, re-irradiation, off-label systemic therapies, and enrollment in clinical trials [71, 120].

The development of immunotherapy has markedly advanced the treatment of many malignancies, including melanoma and various hematologic cancers [64, 73, 117]. Approaches such as oncolytic viruses, immune checkpoint inhibitors, CAR T cells, and antibody-based therapies have each demonstrated clinical benefit in select settings [64, 73, 117]. In contrast, GBM has shown limited responsiveness to these modalities in part due to specific features such as marked tumor heterogeneity, restricted CNS delivery, and a profoundly immunosuppressive tumor microenvironment (TME) that hinders immunotherapy efficacy [18, 126].

The development of effective immunotherapies for GBM is complicated by extensive cellular, genetic, and phenotypic heterogeneity that exists both between patients and spatially within individual tumors and continues to evolve over time [12]. Spatially, potentially targetable antigens exhibit variable expression across tumor regions. For example, in cellular tumor regions, clusters of cells expressing IL13Rα2 and HER2 coexist with other clusters expressing EGFR, whereas in areas of pseudopalisading necrosis, cells expressing these commonly targeted antigens are largely restricted to the borders surrounding hypoxic cores [12]. Additionally, the clustering and intermixing of the various antigen-expressing tumor cells, as well as cell type (mesenchymal, proneural, and classical), can vary within an individual's tumor and between patients [37, 139]. Temporally, therapeutic pressure promotes the emergence of resistant subclones and antigen loss variants [79, 99], contributing to rapid disease progression. Together, these dynamics limit the effectiveness of single-antigen immunotherapies and highlight the potential utility of multi-targeted therapeutic approaches for GBM.

Delivery of anti-cancer therapies in GBM also remains challenging due to the natural barriers keeping the central nervous system (CNS) protected from the rest of the body. Additionally, the highly immunosuppressive, myeloid-dominated GBM microenvironment further constrains immune activity, impairs antigen presentation, and promotes upregulation of checkpoint pathways associated with T-cell dysfunction [42, 54, 108, 113, 132]. Together, these features create an immunologically cold and difficult-to-access TME. Researchers are now developing therapies to equip these inactive immune cells to recognize and kill tumor cells, including engineered antibodies that engage T cells. There have also been promising recent advancements in methods to increase the accessibility of therapies to the CNS. In this review, we examine the biological rationale, preclinical evidence, delivery strategies, and safety considerations supporting the development of T-cell engagers as effective therapeutic agents for GBM.

### T-cell engagers

T-cell engagers are immunotherapeutic agents designed to redirect and activate T cells to target and kill tumor cells. These "off-the-shelf" antibody-based therapies can be manufactured, stored, and distributed much more readily than other cell-based immunotherapeutics, such as CAR T cells, which require autologous, patient-specific engineering that is labor and time intensive [43, 58]. By design, T-cell engagers hold potential to address both tumor antigen heterogeneity and the immunosuppressive nature of the TME. This occurs by directly activating a T-cell through binding to a surface receptor, most commonly CD3, thereby turning “on” the inactive T-cell and linking it to one or more tumor-associated antigens (TAAs) on cancer cells. This physical bridge between the T cell and tumor cell enables efficient T cell-mediated killing of tumor cells by granzyme and perforin release, as well as the activation of other immune cells through cytokine release [85, 128, 138]. In doing so, T-cell engagers can bypass antigen presentation to induce MHC class-independent cytotoxicity, enabling immunosuppressed T cells in the TME to be reactivated and to target cancer cells, as well as aiding in the recruitment and activation of T cells from the periphery into the TME to help kill tumor cells [22].

**Fig. 1:**
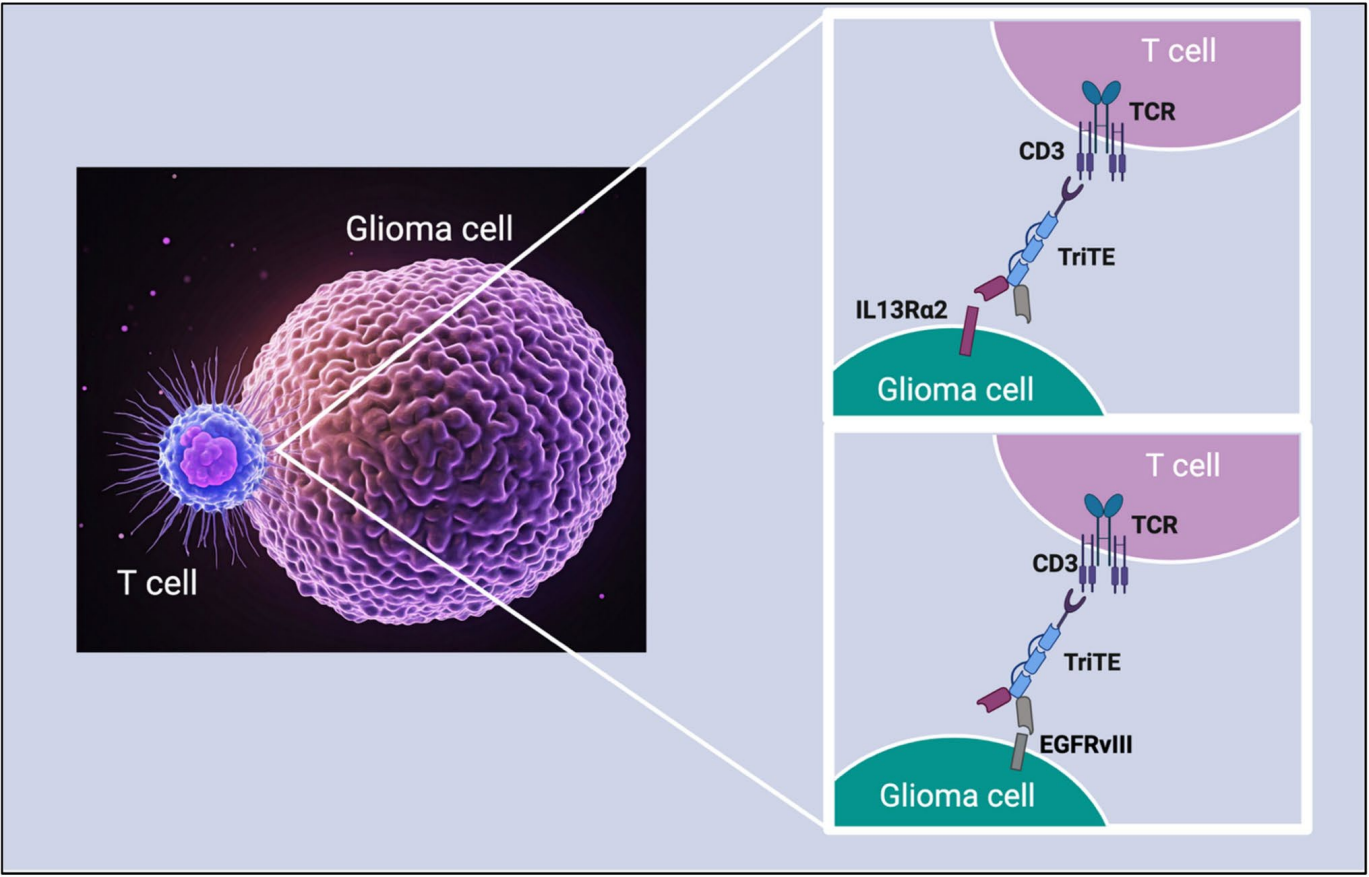
Design of trispecific antibodies (TriTEs). TriTEs are constructed using a modular approach, combining building blocks such as scFv, Fab, or single-domain antibodies to target three distinct antigens. The construct also contains a linker, which determines TriTE's flexibility and stability. The ability of TriTEs to target multiple tumor antigens or pathways can help address the complexity of the GBM tumor microenvironment. *CD3* cluster of differentiation 3, *EGFRvIII* epidermal growth factor receptor variant III, *Fab* fragment antigen binding, *IL13Rα2* interleukin-13 receptor subunit alpha 2, *scFv* single-chain variable fragment, *TCR* T-cell receptor, *TriTE* trispecific T-cell engager. Created with BioRender.com

T-cell engagers can be designed to target two or even three antigens, termed bispecific T-cell engagers (“BTEs”) or trispecific T-cell engagers (“TriTEs”), respectively. While BTEs against single antigens such as IL13Rα2, EGFRvIII, HER2, and B7-H3 have demonstrated preclinical efficacy in GBM models, their potential for limited durability due to antigen loss restricts their clinical applicability [18, 118]. TriTEs address these limitations by simultaneously engaging two TAAs within a single construct (Fig. 1). By targeting multiple antigens, TriTEs hold the potential to distribute selective pressure across diverse tumor cell populations, thereby reducing the emergence of antigen-negative subclones and the risk of disease recurrence. Furthermore, the integration of co-stimulatory or checkpoint-blocking domains extends in vivo therapeutic persistence, improves tumor regression, and further mitigates antigen-negative escape [22, 36, 110].

## Designing TriTEs to target GBM

### Selecting antigen targets

Effective TriTE design for GBM requires careful antigen selection that balances tumor specificity, biological relevance, and antibody-based accessibility. Ideal antigens are highly expressed on tumor cells, minimally present in normal brain tissue, and functionally linked to tumor growth, survival, or immune evasion. Among the most extensively validated GBM-associated or specific antigens are IL13Rα2, EGFRvIII, EGFR, HER2, and B7-H3. IL13Rα2 is expressed on approximately 60–75% of GBMs and is largely absent from normal brain tissue. Activation of the IL13 receptor activates signaling mediators such as (Src, Akt, Erk, Fak) to promote invasion, proliferation, and survival [13, 14, 19, 143]. EGFRvIII, a constitutively active deletion mutant of wild-type EGFR, is present in ~ 30% of GBMs and drives oncogenic signaling that enhances proliferation, metabolic adaptability, and treatment resistance in cancer cells, while remaining undetected in healthy cells [5, 122]. Wild-type EGFR is also frequently overexpressed in ~ 40–60% of GBMs and promotes tumor cell proliferation, invasion, and resistance to chemotherapy and radiotherapy [127]. HER2 is overexpressed in up to 80% of GBMs and promotes ligand-independent proliferative signaling, although expression is often heterogeneous and relatively low in secondary GBM tumors [4, 84, 93, 144, 152]. Finally, B7-H3 is upregulated in 70–80% of tumors and contributes to immune evasion and invasive behavior through JAK2/STAT3-dependent pathways [35]. Together, these antigens represent high-confidence targets for immune redirection strategies in GBM.

BTEs targeting these canonical antigens have provided important proof of principle for antigen-restricted immune redirection in GBM. IL13Rα2-directed BTEs have demonstrated robust T-cell activation, effective tumor localization, and a survival benefit in orthotopic patient-derived xenograft models, whether delivered locally, systemically, or via gene-encoded platforms [18, 40, 106]. EGFRvIII-directed BTEs similarly induced tumor regression and intratumoral T-cell infiltration in preclinical models, motivating clinical evaluation of AMG 596 (etevritamab; NCT03296696) with early reports suggesting acceptable tolerability [6, 70]. However, these studies did reveal a central limitation of single-antigen targeting in GBM: antigen-negative escape driven by intratumoral heterogeneity. In a DNA-encoded bispecific tandem engager model, monotherapy targeting HER2 led to tumor recurrence in ~ 90% of cases due to outgrowth of HER2-negative subclones. In contrast, dual targeting of HER2 and EGFRvIII achieved durable tumor clearance in ~ 80% of animals [104]. These findings provide a strong rationale for multispecific designs.

Researchers have therefore expanded beyond canonical GBM antigens to evaluate additional targets supported by glioma biology and engager studies in other malignancies. EpCAM expression correlates with glioma WHO grade, proliferation, and prognosis, suggesting a role in aggressive GBM subsets [27, 74, 78, 111, 128, 146]. CD38 is expressed on a minority (~ 7–9%) of GBM cell lines and may also mark immunosuppressive stromal or myeloid compartments within the tumor microenvironment [86, 97, 140]. Members of the mucin family (including MUC1, MUC12, and MUC16) have been implicated in glioma progression and therapeutic resistance through the PI3K/AKT/STAT3 and EGFR/MAPK/ERK signaling pathways [32, 46, 105]. Prolactin receptor (PRLR) signaling promotes glioma cell proliferation, migration, and chemoresistance [8, 149]. Another emerging GBM-relevant target is the NKG2D ligand family, including MICA/B and ULBP-family ligands, which are broadly, though variably, detected on GBM cells and patient-derived glioma stem-like cultures. Because NKG2DL expression is heterogeneous and stress-inducible, NKG2D-directed engagers may be particularly useful in combination with TMZ, radiation, or other stress-inducing therapies to broaden targeting of therapy-exposed GBM subpopulations [10, 16, 53]. At the same time, prostate-specific membrane antigen (PSMA) is selectively expressed on GBM tumor vasculature and contributes to angiogenesis [55, 68, 82], offering an opportunity to target non-tumor cell compartments. Collectively, these observations support the inclusion of atypical GBM-associated antigens, validated biologically in glioma and therapeutically in other cancers, as complementary targeting arms in TriTE design. Table 1 summarizes target antigens incorporated into T-cell engagers in GBM and other cancers that may inform TriTE design for GBM.

**Table 1:** Summary of BTE antigen targets, grouped by those evaluated in GBM models versus those validated in other cancers with GBM-relevant antigen expression, along with estimated antigen prevalence in GBM, key expression profile features and, where available, associations with molecular/cellular GBM states

Cancer	Antigen	Model	Summary	Prevalence	Expression profile	References
GBM	IL13Rα2	PDX GBM models and GBM glioma lines; orthotopic and subcutaneous mouse models	IL13Rα2-BTEs, delivered via NSCs, DNA-launch vectors, or recombinant protein, consistently activated CD8⁺/CD4⁺ T cells, eradicated implanted GBM tumors in 60–100% of mice, and extended median survival from ~ 30 to > 65 days with minimal off-tumor cytokine release	~ 60–75% of GBM; minimal/absent in normal brain	Often high expression when present, but intratumorally heterogeneous; enriched in mesenchymal GBM; spatially patchy across tumor regions	[12, 18, 19, 40, 103, 106, 138]
	EGFRvIII	GBM PDX spheroids; intracranial and subcutaneous mouse models; early clinical cohort (Phase I)	EGFRvIII-BTEs potently recruited T cells to kill EGFRvIII⁺ GBM cells in vitro, achieved complete remissions in up to 75% of PDX-bearing mice, and showed safety/tolerability in an initial Phase I release before trial termination	~ 20–30% of GBM overall; usually a subset of EGFR-amplified tumors	Patchy/heterogeneous intratumoral distribution; typically associated with EGFR-amplified/classical-type GBM; prone to antigen loss	[5, 6, 41, 56, 67, 70, 104, 118, 130]
	EGFR (wt)	Human GBM cells; intracranial xenograft mouse models; recurrent GBM patients	EGFR-BTE–armed T cells eliminated heterogeneous EGFR⁺/EGFRvIII⁻ GBM in orthotopic models without off-tumor toxicity; in a human study, intraventricular EGFR-BTE–secreting CAR T cells induced rapid tumor regression in recurrent GBM, though responses were transient	~ 40–60% overexpression in GBM	Enriched in classical subtype; present in cellular tumor regions and at borders of pseudopalisading necrosis	[12, 29, 30, 127, 130]
	HER2	Heterogeneous GBM xenografts; stem-like GBM cultures	HER2-BTE–armed T cells lysed HER2⁺ and TMZ-resistant GBM lines, including stem-like populations; dual HER2/EGFRvIII bispecific tandem (DBTE) constructs achieved up to 80% complete tumor clearance in mixed-antigen models and prolonged survival	Detectable in up to ~ 70–80%, depending on assay/cutoff; true high-intensity expression is less common	Common but low/intermediate intensity and heterogeneous staining; lower in secondary GBM; clustered with regional variation across tumor niches	[12, 93, 104, 150]
	B7-H3	Human GBM cells (U87, U251), subcutaneous xenograft model, orthotopic intracranial model	B7-H3-BTEs can effectively target GBM, but durable efficacy requires enhanced delivery or immune amplification. Significant tumor control and extended survival were achieved in optimized intracranial models	High-frequency expression, commonly ~ 70–80% or higher across GBM series	Broad membranous/cytoplasmic tumor cell expression with additional tumor-associated vascular expression; retained in recurrent disease; high coverage anchor target	[35, 48, 89, 148]
	NKG2DL	Human GBM cells (U87, patient-derived glioma stem-like cells)	Synergistic cytotoxicity was observed with NKG2D-directed BTE treatment in combination with the standard-of-care treatment, TMZ + radiation	Broad but variable; no clear GBM-wide % reported; detected in GBM cells and patient-derived GSC-like cultures	Heterogeneous, stress-inducible MICA/B and ULBP-family ligands; present on GBM and GSC-like cells; inducible by HDACi/therapy stress	[10, 16, 23, 53]
Non-GBM	EpCAM	Multiple solid tumors (uterine, ovarian, lung, gastric, pancreatic, CRC, HCC, breast, prostate, bladder)	EpCAM-targeted BTEs/TriTEs elicited robust T-cell activation and selective lysis across diverse carcinomas, overcame intra-tumor heterogeneity, and mediated > 90% tumor reduction in several models, suggesting applicability to EpCAM⁺ GBM subsets	Subset of GBM tumors;** e**xact prevalence varies; expression increases with glioma grade	Membranous/cytoplasmic and heterogeneous; associated with higher grade, proliferation, and worse prognosis rather than a firmly established canonical GBM subtype	[27, 51, 74, 78, 80, 111, 114, 128, 133, 146]
	CD38	Multiple myeloma cell lines; murine MM models; Phase I MM trials	CD38×CD3 IgM-based BTE (Igm-2644) and CD38 TriTEs caused potent MM cell lysis in vitro, suppressed tumor growth in mice, and demonstrated activity in early clinical studies, highlighting potential use against CD38⁺ GBM stromal elements	Low frequency; only a small fraction of tumor cells are positive, with additional expression in stromal/myeloid compartments	Sparse and heterogeneous on tumor cells; more relevant as a microenvironmental/stromal complement with biologic targeting rationale	[39, 69, 86, 97, 140]
	MUC family (MUC1, MUC12, MUC16)	Ovarian and colorectal carcinoma models; adenoviral delivery for MUC1	MUC1-BTE adenovirus (TILT-322) eradicated MUC1⁺ ovarian tumors. MUC12- and MUC16-directed BTEs induced strong, antigen-dependent cytotoxicity and synergized with PD-1 or anti-VEGF agents, indicating potential for MUC⁺ GBM niches	Incompletely defined; subset expression of MUC1 in GBM; MUC12/MUC16 data remain limited	Aberrant MUC1 expression and function in tumor cells; MUC12/MUC16 have limited data and remain exploratory as of now; stronger biological rationale due to role in glioma progression	[15, 32, 75, 105, 137]
	PRLR	PRLR⁺ breast cancer cell lines; xenografts	PRLR-BTE constructs drove T-cell proliferation and Th1 cytokine release, reduced tumor burden in mice, and showed favorable safety, suggesting PRLR⁺ GBM subpopulations could be similarly targeted	Subset expression in GBM; limited data on exact prevalence; one study reported ~ 30%	Tumor cell expression linked to proliferation, migration, and chemoresistance; heterogeneous; no strong consensus subtype label yet	[1, 8, 9, 149]
	PSMA	Prostate cancer cell lines; Phase I pasotuxizumab trial; squamous lung xenografts	PSMA-BTE pasotuxizumab induced dose-dependent declines in PSA and durable responses in patients with prostate cancer, and preclinical lung models confirmed T-cell recruitment. GBM's PSMA⁺ neovasculature can be an attractive new target	Predominantly a vascular target marker; present on tumor neovasculature in a large proportion of cases, with low expression in normal vessels. Some studies suggest ~ 31–100% prevalence	Vascular target, not compartment specific or diffuse across tumor cells; strongest in proliferating tumor-associated endothelium	[28, 55, 65, 68, 82, 88, 142]

*BTE* bispecific T-cell engager, *CAR* chimeric antigen receptor, *CD* cluster of differentiation, *CRC* colorectal cancer, *DBTE* dual bispecific tandem engager, *EGFR* epidermal growth factor receptor, *EGFRvIII* epidermal growth factor receptor variant III, *EpCAM* epithelial cell adhesion molecule, *GBM* glioblastoma, *GSC* glioma stem cell, *HCC* hepatocellular carcinoma, *HDACi* histone deacetylase inhibitors, *HER2* human epidermal growth factor receptor 2, *IgM* immunoglobulin M, *IL13Rα2* interleukin-13 receptor subunit alpha 2, *MICA/B* MHC class I chain-related proteins A and B, *MM* multiple myeloma, *MUC* mucin, *NSC* neural stem cell, *NKG2DL* natural killer group 2 member D ligands, *NSCLC* non-small cell lung cancer, *PD-1* programmed cell death protein 1, *PD-L1* programmed death-ligand 1, *PDX* patient-derived xenograft, PRLR prolactin receptor, *PSA* prostate-specific antigen, *PSMA* prostate-specific membrane antigen, *Th1* T helper type 1, *TMZ* temozolomide, *TriTE* trispecific T-cell engager, *ULBP* UL16-binding protein, *VEGF* vascular endothelial growth factor, *wt* wild type

Importantly, no single GBM antigen exhibits both universal prevalence and spatially uniform expression. IL13Rα2 shows mesenchymal enrichment, EGFR and EGFRvIII are typically linked to EGFR-driven classical-like disease, HER2 is frequently heterogeneous and often low-intensity, and B7-H3, although broadly expressed, still varies across tumor cell and vascular compartments [5, 12, 19, 35, 93, 127, 130]. For the more non-canonical and exploratory antigens, such as EpCAM, CD38, PRLR, and PSMA, the therapeutic opportunity may lie less in pan-tumor coverage and more in targeting aggressive subpopulations or distinct microenvironmental compartments such as neovasculature. This heterogeneity provides a central rationale for multi-targeted T-cell engagers, which can pair broadly expressed antigens with more tumor-specific or compartment-restricted targets to reduce antigen-negative escape (Table 1).

Thus far, only two TriTE constructs have been evaluated directly in GBM models, providing early evidence that multi-antigen engagement can overcome the limitations of bispecific approaches. Park et al. [103] engineered DT2035, a CD3-redirecting DNA-encoded TriTE construct targeting both IL13Rα2 and EGFRvIII. Notably, DT2035 activated CD4⁺ and CD8⁺ T cells from GBM patient-derived peripheral blood mononuclear cells. This elicited a strong granzyme–perforin pathway-mediated response as well as cytokine-mediated cytotoxicity without exacerbating cytokine release syndrome. In heterogeneous intracranial xenografts, DT2035 achieved complete tumor clearance in 100% of mice at day 35 within Balb/c nude mice, and in a long-term survival study of NSG-K mice, nine out of nine mice survived at day 84, and six out of nine mice were still surviving at day 122, whereas monospecific controls produced only partial responses. Of the surviving mice, none showed any weight loss or adverse events [103].

Our laboratory generated a TriTE molecule that targets IL13Rα2 and EGFR/EGFRvIII, with the distinction that a nanobody targeting EGFR/EGFRvIII instead of scFv has been incorporated in the design of the TriTE [11, 91]. When tested in a mouse model that was implanted with PDX lines expressing both antigens (GBM6/GBM39 orthotopic grafts), TriTE enhanced with an sc(Fc)2 domain resulted in a 37-day median survival, which was significantly higher than its BiTE (IL13Rα2/CD3) target (29 days) and control (28 days). This was similarly observed in GBM6-only-implanted mice, confirming that targeting two antigens results in a more robust survival benefit [11, 91].

Despite these promising results, several limitations of the two current GBM-focused TriTE studies must be addressed in future development. DT2035 was evaluated in immunodeficient xenograft models that do not fully capture the complexity of the human BBB or the profoundly immunosuppressive GBM TME. One way this could be addressed is by injecting exhausted or inactive T cells alongside the TriTE constructs to see whether the antibody can “recover” the function of immunosuppressed immune cells, which would better reflect the environment in GBM patients. Additionally, antigen loss-mediated escape was not monitored. It would be interesting to observe animals for a longer period (i.e., closer to the end of their normal lifespan) for any radiographic or histologic recurrence, and evidence of antigen escape. This would be costly, but could provide helpful clinical data on how many doses may be required to achieve tumor eradication in patients.

Beyond IL13Rα2 and EGFRvIII TriTEs, a broader survey of TriTEs in other solid tumors reveals design principles readily translatable to GBM. In colorectal cancer, EGFR×EpCAM×CD3 TriTEs achieved up to 100-fold greater potency and durable tumor inhibition compared with bispecific controls [128]. Kimura et al. developed TaKE1, an EGFR×CD3×CD16 trispecific that recruits both T and NK cells, and demonstrated enhanced cytotoxicity at low effector-to-target ratios [76]. Together, these studies show that TriTEs can be engineered not only to broaden tumor antigen coverage but also to directly engage immune cell pathways, thereby improving the durability of antitumor responses. Most engager formats bind CD3 to provide the primary activation signal for T-cell recruitment and cytotoxicity, but CD3 signaling alone may be insufficient in solid tumors. Accordingly, newer multispecific constructs have incorporated conditional CD28 costimulation to provide the second signal required for full T-cell activation, as well as PD-1/PD-L1 blockade to restore T-cell activation in exhausted cells [21, 109, 140]. In some cases, checkpoint blockade has been paired with conditional 4-1BB agonism or IL-6R targeting to integrate immune activation, reversal of exhaustion, and cytokine-risk control within a single molecule [22, 110]. Because systemic activation of pathways, such as CD28, is associated with severe toxicity, these designs typically aim to restrict immune stimulation to the tumor site [21, 109, 140]. Such immune cell-directed TriTE strategies can not only help overcome antigen heterogeneity but also restore durable effector function within the GBM tumor.

### Choice of antibody fragments and inter-domain linkers

In addition to target antigens, the performance of T-cell engagers is strongly influenced by their molecular architecture. Natural antibodies contain a constant domain (Fc) and two antigen-binding sites, each with a heavy (H) and a light (L) variable domain [81, 107]. Designing a bispecific construct that incorporates two distinct antigen-binding sites poses a technical challenge due to the “chain-association issue”. In protein assembly, various combinations of the VH and VL chains can associate, ultimately leading to inconsistent generation of the target result (two separate antigen-binding sites), since only a specific VH–VL combination will yield that endpoint. To avoid this, the field has instead transitioned to fragment-based constructs, such as tandem scFvs, which allow customization and specification of the desired result. These formats, however, are generated without the glycosylated constant region, resulting in a relatively short plasma half-life because the Fc region protects against catabolism. Most antibodies tested in glioblastoma have been tandem scFvs (Table 2). An alternative design would be to fuse a regular antibody molecule to 1–2 polypeptide chains, thereby preserving the Fc domain. This design, however, results in steric hindrance, especially when trying to engage two cell types simultaneously (both cells would be on the ipsilateral side of the Y-shaped antibody). This has led to asymmetric alternatives that seek to retain the native architecture of antibodies, with antigen-targeting sequences at distant sites on the Y-shaped antibody, using complex manufacturing techniques such as post-production assembly or the recombination of antibody half-molecules to generate the desired result. The construct aims to reduce immunogenicity and side effects by closely resembling natural antibodies; however, its asymmetric nature typically limits targeting to only two antigens, thereby preventing multivalent targeting of 3+ antigens. Additionally, a concern regarding including the Fc domain is cytokine release syndrome (CRS). When the anti-human CD3 antibody (OKT3), which contains an Fc region, was first tested in humans, it resulted in severe CRS involving the Fc regions of CD3 antibodies and Fc receptors on other immune cells [25]. Therefore, to reduce off-target toxicities, many of the antibodies currently being tested have been engineered without the Fc domain (Table 2), except for the B7-H3/CD3 model by Liu et al., which utilized dual-affinity re-targeting technology to preserve the Fc domain, and our laboratory's TriTE construct against IL13Rα2 and EGFRvIII. The construct generated by Liu et al. resulted in a prolonged half-life of the antibody, with retention of up to 120 h post-injection [89]. Our laboratory demonstrated an extended plasma half-life for the TriTE construct containing the Fc domain [11, 91]. However, the risk of cytokine release syndrome was not directly measured in either study. It would be beneficial in future studies to directly compare a construct with and without an Fc domain in an immunocompetent host regarding its bioavailability and toxicity profile before initiating clinical trials.

**Table 2:** Summary of T-cell engager construct designs and comparison of efficacy of each format within GBM models

Target antigen	Target antigen antibody type	Linker	T-cell antigen	T-cell antigen antibody type	Efficacy	References
IL13Rα2	scFv	GS	CD3	scFv-forward orientation (V_L_–V_H_–V_H_–V_L_)	Lower CD3 affinity but no off-target toxicity compared to reverse orientation; 100% tumor growth rejection and survival at 55 days, crosses the BBB	[18]
	scFv (Hu08)	GS	CD3	scFv-forward orientation	High target specificity and no off-target toxicity when co-cultured with the 5077 cell line without IL13 mutation; tumor burden significantly decreased in IL13Rα2+ U87MG implanted mice [BTE 30 times more effective than CAR T counterparts)	[138]
	scFv of mAb47	GS-short (5 aa) vs long (23 aa)	CD3	scFv of mAb OKT3 (V_H_–V_L_–V_L_–V_H_)	Long linker with 8.5 × higher affinity than short linker. Long linker resulted in increased survival of mice (63–67% increase in GBM6 and GBM12 models, respectively)	[106]
IL13Rα2 and EGFRvIII	scFv	GS	CD3	scFv—tested in three different orientations with respect to scFv tumor fragments, central, N-terminus, and C-terminus	All constructs demonstrated strong binding to target cells in vitro, with central CD3 showing slightly increased activity. Central CD3 TriTE demonstrated 100% survival in the mouse model at day 35. In extended survival studies, 9/9 mice survived up to 84 days, and 6/9 survived up to 122 days	[103]
	scFv (IL13Rα2); nanobody (EGFR/EGFRvIII)	GS	CD3	scFv	TriTE construct with an Fc domain was made in a 5′–3′ orientation with CD3 scFv VH–(G_4_S)_3_–CD3 scFv VL–(GGGSGGGGSGSGGGSGGGGSGGG)– IL13Rα2 scFv VL–(G_4_S)_3_–IL13Rα2 scFv VH–first CH2–CH3 region of Fc–(G_4_S)_6_–second CH2–CH3 region–(G_4_S)_3_–EGFRvIII nanobody. TriTE demonstrated significant on-target killing of the GBM6 cell line, which is positive for IL13Rα2 and EGFRvIII, and the GBM39 cell line, which is positive for EGFRvIII. GBM6/GBM39-implanted mice treated with TriTE had a median survival of 37 days, significantly longer than that of the BTE (IL13Rα2/CD3) target (29 days) and the control (28 days). This was similarly observed in GBM6-only-implanted mice	[11, 91]
EGFRvIII	scFv (806)	GS	CD3	scFv- forward orientation	High target specificity, high T-cell activation (CD4 and CD8) with cytokine production, showed dose-dependent targeting of EGFR in vitro	[138]
	scFv at N-terminus	Short peptide (unspecified)	CD3	scFv at C-terminus	40–73% of mice survived with 0.1–0.5 mg/kg of BTE at 44 days. No signs of toxicity were found after testing in three cynomolgus monkeys, including any signs of cytokine storm	[118]
	Two Fab heterodimeric format	Unspecified	CD3	Fab	Bivalent binding to the target antigen significantly reduced tumor volume and increased T-cell infiltration in GBM orthotopic models	[70]
	scFv	GS	CD3 (clone UCHT-1)	scFv	100% (*n* = 10) orthotopic mice demonstrated tumor burden clearance; did not inhibit selective killing of HER2+ tumors when co-administered with an anti-HER2 BTE	[104]
	scFv (806)	GS	CD3	scFv (OKT3)	No significant survival benefit despite an increase in cytotoxicity in vitro compared to controls; this remained the same even with co-treatment of macrophages designed to secrete IL12	[56]
	scFv (R6)	Unspecified	CD3	scFv (OKT3)	Complete tumor regression in mice co-treated with immunotoxin and BTE	[67]
	scFv	GS	CD3	scFv	When produced within a CAR T format, BTE resulted in significant tumor toxicity across multiple EGFR+ cell lines (U87, U251) and U251 mice; did not have any off-target cytotoxicity	[30]
HER2	scFv	GS	CD3 (clone UCHT-1)	scFv	Co-administration of BTE against HER2 and EGFRvIII showed greatest survival (80% survival at day 34 with the remaining 20% demonstrating tumor antigen escape)	[104]
	mAb (trastuzumab)	Unspecified	CD3	scFv (OKT3)	Armed T cells with BTE exhibited 50–80% cytotoxicity against four different glioma cell lines as well as a TMZ-resistant variant of U251	[150]
B7-H3	VHH	Mouse IgG2a Fc	CD3 and IL15–15Rα	scFv	Immunocytokine composed of a BTE fused to the IL-15/IL-15R domain resulted in an increase in T-cell and NK cell recruitment into the TME, resulting in significantly more T- and NK cell activation and killing of tumors	[148]
	scFv (VH–linker–VL)	GS	CD3	scFv (VH–VL)	Resulted in prolonged survival benefit when combined with dEGCG and IFN-γ, which were added to the construct to aid in the production of reactive oxygen species and ferroptosis	[48]
	Fv (includes Fc)	Peptide linker	CD3	Fv (includes Fc)	Utilized dual-affinity re-targeting technology, which facilitates heterodimerization and stable linking between cancer and T cell; prolonged half-life of the BTE with retention up to 120 h post-injection	[89]
NKG2D	Ectodomain (amino acids 78–216)	GS	CD3	scFv	Treatment with BTE resulted in significantly increased in vitro killing compared to control groups. This was enhanced by the addition of TMZ, radiation, and TMZ + radiation subgroups	[16]

*IL13Rα2* interleukin 13 receptor alpha 2, *scFv* single-chain variable fragment, *GS* glycine-serine, *CD* cluster of differentiation, *V*_*L*_ variable light chain, *V*_*H*_ variable heavy chain, *BBB* blood–brain barrier, *BTE* bispecific T-cell engager, *CAR T* chimeric antigen receptor T cell, *mAb* monoclonal antibody, *EGFRvIII* epidermal growth factor receptor III, Fab antibody fragment, *HER2* human epidermal growth factor receptor 2, *TriTE* trispecific T-cell engager, *Fc* antibody constant fragment, *TMZ* temozolomide, *VHH* variable domains of heavy-chain antibody, *NKG2D* natural killer group 2 member D

Another consideration is the type of linker between the tumor and immune cell antigen fragments. Almost all constructs used a glycine-serine (GS) linker, which allows high flexibility given its small side chain, thereby conferring maximal backbone conformational freedom [24]. This flexibility allows for proper VH–VL pairing within the scFv domains, permitting the two binding arms (anti-CD3 and antitumor antigen) to orient independently for target engagement. The GS linker is one of the smallest peptide linkers, helping reduce the overall molecular size burden, an important consideration in designing therapeutics that could cross the BBB. It also reduces the distance between the immune cell and its target, thereby increasing cytotoxic potency. However, given its repetition, it may also generate higher immunogenicity when longer GS linker sequences are used. Excessive flexibility may result in antibody fragments coiling on one another, leading to less stable immunological synapses and reduced cytotoxic efficacy [77, 83]. Pituch and Zannikou et al. demonstrated the superior functionality of BTE with the 22-amino acid GS linker compared to BTE with the 5-amino acid length that many other constructs tested within GBM have utilized [106]. They noticed an 8.5 times higher affinity as well as a 63–67% increase in survival in mice compared to the shorter linker configuration [106]. This study suggests a Goldilocks effect: the linker needs to be just long enough to facilitate immune cell–tumor cell interaction, but not too long for the antibody to coil up on itself and create unstable immunologic synapses.

When specifically considering TriTE molecules, an additional consideration concerns the orientation of the CD3 fragment relative to the tumor antigen fragments. Park et al. tested three configurations of CD3 positioning relative to the IL13Rα2 and EGFRvIII domains and found that centralizing the CD3 binding domain optimized the immunological synapse geometry and minimized off-target engagement [103]. Our laboratory designed an asymmetric TriTE construct that arranged the CD3 antibody fragment as the first sequence, followed by an IL13Rα2 fragment, then the Fc domain, and finally the nanobody targeting EGFRvIII [11, 91]. We found that the TriTE protein had a half-life of 41 h, could cross the BBB, and conferred a significant survival benefit when tested in GBM6- and GBM6/GBM39-implanted mice. When comparing these two constructs, the TriTE with the CD3 domain placed between the two tumor antigen-binding fragments showed a greater survival benefit. However, the comparison is limited by the use of different animal models (Park et al. used U87vIII and U251 orthotopic mice, whereas Balyasnikova et al. used GBM6 and GBM39 orthotopic mice). Taken together, the studies suggest that while all orientations appear efficacious, customizing the construct to unique binding arms used in TriTE's design is imperative for selecting the lead design.

## Delivery considerations

When considering CNS tumors such as GBM, there are three barriers to systemically delivered immunotherapies that need to be overcome: the blood–brain barrier (BBB), blood–brain tumor barrier (BTB), and blood–CSF barrier (BCSFB) [72]. The BBB classically limits the penetration of large, hydrophilic molecules, including antibodies and engineered immune cells, into healthy brain parenchyma, severely restricting therapeutic delivery [3]. In GBM tumors, the BTB is an altered BBB, as it is overall leakier due to increased abnormal, tortuous blood vessels feeding the tumor, aberrant pericyte distribution, and loss of astrocytic foot processes [3, 72]. Since the BTB is generally less selective than the BBB, some drugs may have an easier time entering the tumor; however, glioblastoma tumors have evolved to evade this by incorporating drug efflux systems [10, 45], posing a significant challenge to drug development. Finally, the last barrier to consider for immunotherapeutic drug delivery is the BCSFB, where the drug would need to bypass the choroid plexus, a tight formation of fenestrated capillaries inside the ventricles [63]. Several enabling approaches are being investigated to tackle these problems.

First, it is important to understand the baseline ability of T-cell engagers in reaching the tumor site when delivered systemically. BTEs approaching 60 kDa have demonstrated intracranial tumor accumulation in GBM despite any interventions to augment their accessibility to the tumor bed. In a longitudinal PET/CT study of a fully humanized IL13Rα2×CD3 BTE, intravenous administration produced rapid tumor localization at 3 h post-infusion, and tumor uptake was on average twice as great as in healthy brain tissue (healthy brain: 0.99% ± 0.41% ID/g vs tumor: 2.03% ± 1.18% ID/g, n = 10, *p* = 0.022). Overall, BTE accumulated heterogeneously throughout the tumor and persisted for at least 24 h, clearing around the 48-h time point. In terms of accessibility, the authors found that the BTE traversed the BTB; however, they noted that further investigation into whether the drug could cross the intact BBB or the BCSFB is needed, especially given that GBM tends to recur in intact areas of the BBB, and knowing whether the drug could penetrate these more heavily guarded areas could help predict therapeutic persistence [50, 101].

Emerging preclinical data from our laboratory suggest that recombinant TriTE proteins may also achieve meaningful tumor accumulation. Our TriTE targeting IL13Rα2, EGFR/EGFRvIII, and CD3 exhibited an extended serum half-life and heterogeneous intratumoral uptake after systemic injection. No additional methods were specifically used to augment trafficking into TME [91]. Park et al. (2024), who also tested a TriTE in GBM, reported significantly prolonged survival in mice treated with TriTE; however, they used electroporation to enhance trafficking to the TME. The exact biomechanics of drug delivery were not elaborated upon, but one can suspect that intratumoral accumulation occurred given the demonstrated survival benefit; to what degree, with or without electroporation, would be interesting for further investigation [103].

Numerous strategies could be employed to enhance delivery of T-cell engagers to the CNS tumor bed. First, radiation treatment has been shown to disrupt the BBB in and around tumors, thereby enhancing the effectiveness of therapeutic delivery [62]. As part of the gold standard treatment of GBM, radiation therapy before or concurrent with BTE may represent an easily translatable option for patients. Baugh et al. recently published a paper demonstrating that their NKG2D ligand-targeting BTE had enhanced activity when pre-treated with TMZ, radiation, or TMZ + radiation, compared with BTE alone [16]. The authors used a radiation dose of 2 Gy in U87 cancer cell lines. While pre-treatment with radiation did show significant improvement in BTE killing of U87 cells, TMZ and RT-TMZ did show greater enhancement of the BTE treatment. Although not assessed in this paper, the improved efficacy of the therapy may have resulted from disruption of the BBB. We suggest that future studies consider testing T-cell engagers with and without radiation treatment and focus not only on assessing efficacy but also on evaluating drug trafficking into the TME.

An additional technique to aid T-cell engagers in reaching the CNS is to directly open the endovascular barriers. Focused ultrasound uses sonoporation via targeted ultrasound waves and intravenously injected microbubbles to open the BBB/BTB in localized brain regions for a transient period of time [7, 20], to increase the ability of large drugs such as TriTEs to cross the BBB/BTB. This has been tested to enhance delivery of immunotherapeutics in preclinical trials [26, 47, 115], and is currently being tested alongside anti-EGFR bispecific-armed T cells in a phase 1 clinical trial at the University of Virginia (NCT07343986) with the study estimated to complete in December 2028.

Encapsulation of the T-cell engager within lipid or polymer nanoparticles is another method that can protect against rapid clearance and peripheral degradation as well as facilitate BBB crossing through endocytosis [48, 59]. Alternatively, BBB shuttle peptides can conjugate TriTEs to ligands or peptides that bind transport receptors on brain endothelial cells (e.g., the transferrin receptor), thereby enabling receptor-mediated transcytosis [112, 124, 147]. These methods may provide particular benefit again for the larger TriTE molecules, hopefully extending the therapeutic benefit of these immunologic agents.

Locoregional delivery into the CSF space can bypass the BBB/BTB, thereby increasing drug concentration in the tumor bed. Intrathecal administration may also reduce systemic toxicity, as therapeutically effective CNS concentrations might be achieved at substantially lower total doses [66, 95, 134]. Locoregional delivery, though, has important limitations, including the need for implanted devices such as an Ommaya reservoir, which introduces device-related risks, including infection [92, 125]. However, given the poor prognosis of this patient population, the authors believe that the potential benefits of locoregional delivery outweigh these risks. In addition, preclinical testing of intrathecal strategies with T-cell engagers is now facilitated by platforms that enable repetitive intrathecal delivery, thereby mimicking the function of an Ommaya reservoir in animal models [145]. With this new system now available, we highly recommend that preclinical studies include comparisons of intrathecal administration with the traditional intravenous, intratumoral, or intraperitoneal routes.

Outside of changing the delivery route or manipulating the BBB itself, an alternative strategy is to encode the T-cell engager into a smaller carrier system that can produce the protein locally. This can be done with gene-encoded delivery, in which plasmid DNA, mRNA, or viral vectors drive in situ production of T-cell engagers. DNA-encoded BTEs delivered via nonviral plasmids or adeno-associated viral vectors have achieved sustained intratumoral expression over days to weeks, reducing the pharmacokinetic fluctuations associated with repeated protein infusions [15, 18, 151]. Safety can be built in through inducible promoters or “on/off” switches, allowing clinicians to dial expression up or down to manage toxicity [34, 38, 60]. Delivery of the DT2035 construct was carried out via DNA-launched expression. Intramuscular administration of a plasmid encoding the TriTE under a constitutive promoter produced sustained intratumoral levels without continuous protein infusion. This gene-encoded format incorporated safety switches and simplified manufacturing [103].

Cellular carriers, which can be modified ex vivo to secrete T-cell engagers and subsequently infused into the brain, are another option for delivery [30, 56, 106]. Neural stem cells engineered to secrete IL13Rα2-targeted BTEs exhibited tumor-tropic migration, drove intratumoral T-cell activation, and improved survival in orthotopic GBM PDX models, supporting their role as local drug delivery platforms in the CNS [56, 106]. CAR T cells represent a second cellular carrier opportunity, with the added advantage of antigen-guided delivery, antigen-dependent expansion, and sustained persistence within the tumor microenvironment. Preclinical and early clinical studies using EGFRvIII-directed CAR T cells engineered to secrete EGFR-targeted BiTE/TEAM molecules demonstrated robust GBM control [29, 30]. Genetically engineered macrophages offer a third carrier platform, as macrophages can traffic to intracranial tumors following systemic administration and accumulate within GBM lesions in response to tumor-derived chemokines. Preclinical studies demonstrate preferential homing of infused macrophages to orthotopic GBM tumors with deep tissue penetration, while early clinical experience with autologous macrophage-based therapies suggests favorable safety and low immunogenicity [31, 49, 52, 102]. However, tumor-associated macrophages undergo continuous turnover over weeks rather than durable engraftment, potentially limiting the duration of therapeutic payload delivery [57]. Along with that, other trade-offs for cellular carriers include the need for invasive delivery methods and the logistical complexity of large-scale manufacturing for patient-specific cell isolation, genetic engineering, expansion, and the consistent production of T-cell engagers.

An additional interesting technique is intein-mediated protein trans-splicing (PTS), in which two separate polypeptide precursors, each carrying different antigen-binding domains and split intein halves, are independently expressed and purified. Upon mixing, the inteins catalyze traceless peptide bond formation to generate a fully functional T-cell engager [123, 133]. PTS reduces aggregation by keeping individual domains apart until final assembly, making it particularly well suited for bulky TriTE constructs. When applied in vivo, successful TriTE formation would require that both protein components reach the TME and co-localize at appropriate stoichiometric ratios to enable productive trans-splicing.

Lastly, the circulation time of T-cell engagers can be extended by genetically fusing them to an immunoglobulin Fc domain (as described previously) or to human serum albumin (HSA), thereby increasing the likelihood that the drug reaches the TME. Both Fc and albumin engage the neonatal Fc receptor, which rescues internalized proteins from lysosomal degradation and recycles them back into circulation. By using this pathway, Fc- or HSA-tagged TriTEs can achieve serum half-lives of days rather than hours, reducing the need for continuous infusion [2, 87]. Both Liu et al. and our laboratory have designed antibodies using this method (Table 2), thereby extending the construct's half-life to 41–120 h.

Overall, each delivery method offers a distinct balance of benefits and drawbacks. For GBM, the optimal approach may combine strategies, such as gene-encoded expression in intracranially delivered NSCs to enhance stability and tumor localization, with a customized approach tailored to a patient's biomarker profile and the pharmacodynamic requirements of the selected TriTE construct.

## Toxicity evaluation

Because T-cell engagers induce potent immune activation within the CNS, careful preclinical toxicity evaluation is essential, particularly to assess the risks of cytokine release syndrome (CRS) and neurotoxicity. Although CRS and immune effector cell–associated neurotoxicity syndrome (ICANS) are well described in hematological malignancies, emerging evidence suggests that GBM-directed T-cell engagers targeting tumor-restricted antigens may induce spatially confined immune activation within the solid tumor microenvironment [94]. In an immunocompetent murine GBM model, a CD3×IL13Rα2 BTE selectively activated tumor-infiltrating CD8⁺ T cells, with no detectable activation of peripheral or splenic T cells; single-cell RNA sequencing confirmed tumor-restricted expansion of effector and tissue-resident memory T-cell populations [141]. These findings suggest that antigen-dependent, tumor-restricted engagement may reduce systemic toxicity risk in GBM patients, unlike those with blood-borne cancer.

Several design and testing strategies can further reduce T-cell engager toxicity. One strategy is step-up dosing, where incremental dose escalation in early treatment phases blunts peak T-cell activation at each step and thereby mitigates peak cytokine responses [96]. Serial monitoring of cytokines in serum and CSF enables real-time assessment of inflammatory responses and identification of dose ranges that balance efficacy with safety [61]. Furthermore, incorporating mutated Fc regions or FcγR-silencing mutations prevents unwanted immune activation [22, 44, 90, 136]. Also, embedding co-stimulatory (CD28, 4-1BB) or checkpoint-blocking (PD-L1) modules can promote localized T-cell amplification and cytokine release at the tumor site [21, 109, 135]. One interesting method is cytokine-profiling assays to select constructs with maximal cytotoxicity yet minimal off-target inflammation. This method involves co-culturing multiple TriTE variants with PBMCs at varying antigen densities and measuring cytokine secretion. This was exemplified in DT2035's early validation [103] and could be used in future testing of upcoming T-cell engagers before clinical trials.

Finally, each TriTE construct should undergo specificity profiling to prevent off-tumor toxicity, including against cells expressing homologous antigens (e.g., wild-type EGFR in EGFR/EGFRvIII-targeted arms) and in healthy brain tissue, to ensure that TriTE-mediated T-cell activation occurs preferentially in tumor tissues. In testing this at the bench, we hope to avoid devastating consequences for patients once the drug starts clinical trials.

## Conclusion

Glioblastoma's antigenic heterogeneity, profoundly immunosuppressive tumor microenvironment, and restrictive delivery barriers have limited the effectiveness of most immunotherapies to date. By distributing therapeutic pressure across multiple antigens and extending bioavailability through recent biotechnological advances, T-cell engagers are emerging as promising molecules to limit antigen-negative escape and improve long-term tumor control. Advances in spatial and single-cell transcriptomics now enable more precise identification of co-expressed antigens within GBM niches, making biomarker-driven patient selection and tailored construct design increasingly feasible. In combination with emerging delivery methods such as PTS or FUS, T-cell engagers may represent an important next step in GBM immunotherapy.

**Fig. 2:**
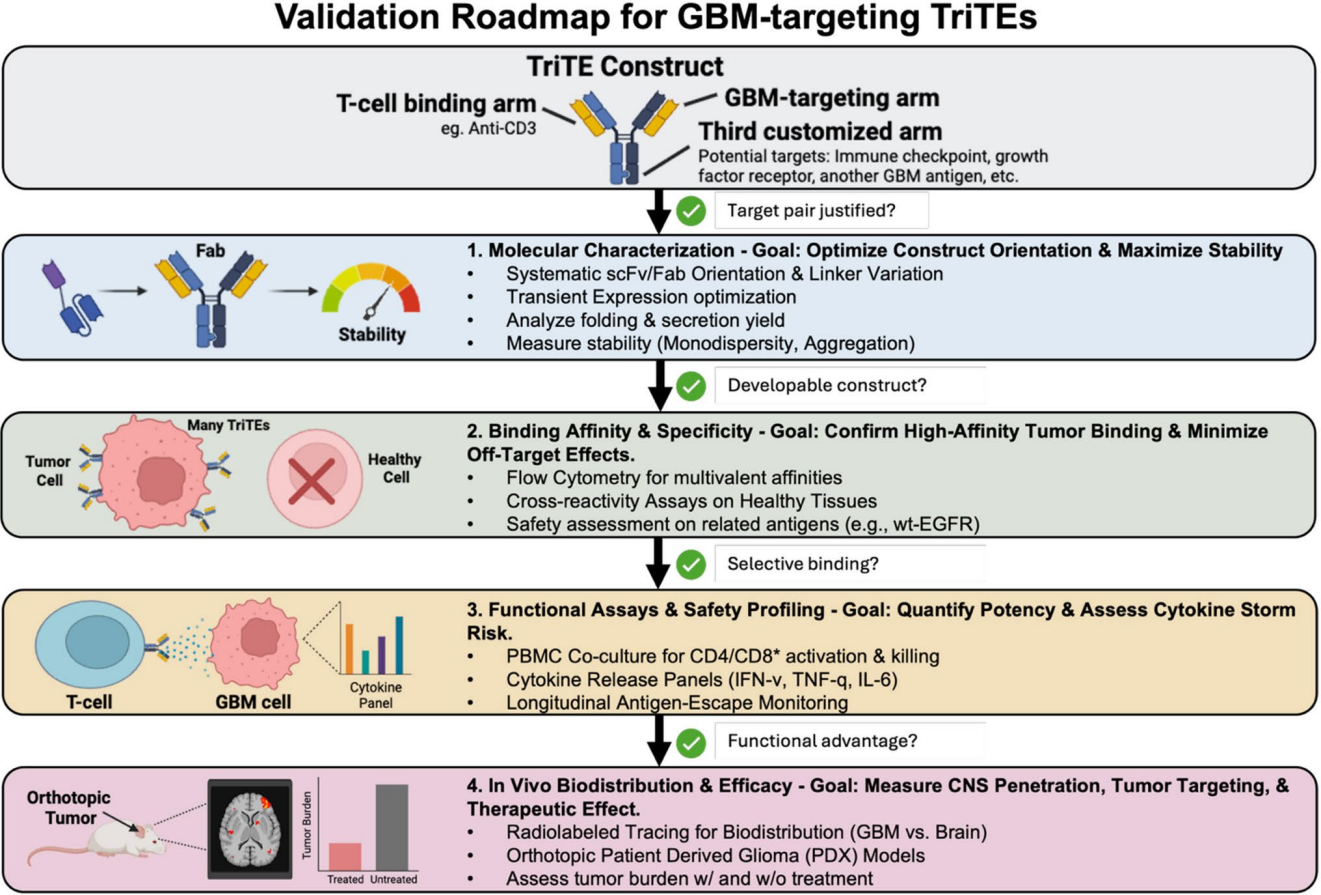
Validation roadmap for GBM-targeting TriTEs. Proposed preclinical validation model, spanning construct design, molecular optimization, target binding, functional safety testing, and in vivo efficacy assessment. Decision checkpoints between boxes indicate that constructs advance only if they demonstrate sufficient antigen-pair rationale, developable molecular architecture, selective target antigen binding, antigen-dependent cytotoxicity across heterogeneous GBM models, acceptable cytokine and off-tumor safety profiles, and in vivo tumor exposure with therapeutic benefit. Failure at any checkpoint should trigger redesign and reconsideration of the previous step. *CD* cluster of differentiation, *CNS* central nervous system, *Fab* fragment antigen binding, *GBM* glioblastoma, *IFN-γ* interferon gamma, *IL-6* interleukin-6, *PBMC* peripheral blood mononuclear cell, *PDX* patient-derived xenograft, *scFv* single-chain variable fragment, *TNF-α* tumor necrosis factor alpha, *TriTE* trispecific T-cell engager. Created with BioRender.com

Clinical translation will require more precise patient selection and construct matching, as GBM exhibits patchy spatial co-expression of targetable antigens and evolves under treatment pressure. As such, it is likely the most effective TriTE design will differ across patients and across disease stages. Emerging tools such as spatial transcriptomics and liquid biopsy-based monitoring may help identify antigen combinations most relevant to an individual tumor and track how those profiles change over time [129, 131]. Although this degree of biomarker-driven tailoring could increase development and manufacturing complexity, it offers the potential to improve therapeutic efficiency by reducing exposure to ineffective constructs. Realizing this potential will require a rigorous translational framework for preclinical validation (Fig. 2). Rather than relying solely on cytotoxicity data, GBM-directed TriTEs will need to progress through an integrated pipeline that links rational design, preclinical validation, and robust clinical trials testing to support clinical translation. A summary of recent or ongoing clinical trials of T-cell engagers is provided in Table 3.

**Table 3:** Summary of recent or currently ongoing clinical trials of T-cell engagers in glioblastoma patients

Trial phase	Trial design	Target antigen	Delivery route	Delivery schedule	Additional therapies?	Patient population	Preliminary efficacy	Safety	Trial dates	NCT
Phase 1	Open label Key inclusion criteria: histologically confirmed newly diagnosed GBM, KPS ≥ 60 Key exclusion criteria: Patients with LMD or subependymal spread or metastatic disease, recent stroke or MI in the past year, received any treatment outside of surgery for GBM	EGFR	IV-infused armed T cells	8 weekly injections	Radiation + TMZ	10 newly diagnosed patients with astrocytoma grade 4 at the University of Virginia	Increased glioma-specific antitumor cytotoxicity by PBMCs (*p* < 0.03) and NK cell activity (*p* < 0.002) ex vivo. Increased serum concentrations of IFN-y (*p* < 0.03), IL-2 (*p* < 0.007), and GM-CSF (*p* < 0.009) Although not primary aim, median PFS was 17.2 months and median OS was 28.8 months. All patients progressed and died from their disease	Highest feasible dose was 80 × 109 without dose-limiting toxicities but rather limited by T-cell expansion	October 2017–January 2020	03344250
Phase 1	Open label Key inclusion criteria: DLL3-positive tumors, documented progression after radiation and/or chemotherapy by RANO criteria, KPS ≥ 70 Key exclusion criteria: presence of metastatic or LMD, prior treatment with bevacizumab or other anti-angiogenic treatment	DLL3	IV infusion	Dose escalation	No	Estimated 20 patients with DLL3-positive tumors at international medical centers including the USA	Unavailable	Results unavailable; primary outcome is the occurrence of dose-limiting toxicity during the maximum tolerated dose (MTD) evaluation period of up to 4 weeks and the entire treatment period of up to 26 months	January 2024–August 2027	05916313
Phase 1 Phase 2	Open label Key inclusion criteria: histologically confirmed glioblastoma or gliosarcoma, prior resection, radiation, and/or chemotherapy, KPS ≥ 70 Key exclusion criteria: severe ICP, status epilepticus or serious complications of the brain tumor requiring urgent intervention, extracranial metastasis, recent MI in the past year or history of CAD requiring medications or evidence of heart failure	EGFR	Intrathecal injections via lumbar puncture of armed T cells	8 biweekly injections over 4 weeks	No	N/a	N/a	N/a	Terminated early due to lack of funding and the primary co-investigator of the trial leaving the institution	02521090
Phase 1	Open label Key inclusion criteria: pathology confirming EGFR-positive glioblastoma or gliosarcoma, IDH wild-type and MGMT unmethylated tumors, KPS ≥ 70, underwent maximal safe resection, non-eloquent BBB opening target Key exclusion criteria: cerebellar or brainstem tumors, evidence of LMD or metastatic disease, recent stroke or MI within 1 year, CAD requiring treatment or evidence of heart failure, received any other therapy outside of surgery	EGFR	IV-infused armed T cells	8 weekly infusions	Delivered with focused ultrasound to improve BBB permeability	Planned for 12 total patients at the University of Virginia; newly diagnosed MGMT unmethylated IDH-WT glioblastoma	N/a	N/a	March 2026–December 2028	07343986
Phase 1	Open label Key inclusion criteria: WHO grade 4 GBM with confirmed EGFRvIII mutation, must have undergone prior resection and concomitant RT and TMZ with adjuvant 6 cycles of TMZ, KPS ≥ 70 Key exclusion criteria: Known HIV or HBV or HCV infection, on ≥ 2 mg dexamethasone daily or equivalent within 7 days of first infusion, active autoimmune disease	EGFRvIII	Continuous IV infusion over 4 days	5 cycles with each cycle being a total of 28 days	No	18 proposed patients at Duke University	N/a	N/a	April 2026–December 2027	04903795

*TMZ* temozolomide, *GBM* glioblastoma, *KPS* Karnofsky performance status, *LMD* leptomeningeal disease, *MI* myocardial infarction, *PBMCs* peripheral blood mononuclear cells, *NK cell* natural killer cell, *PFS* progression-free survival, *OS* overall survival, *RANO* Response Assessment in Neuro-Oncology criteria, *CAD* coronary artery disease, *RT* radiation treatment, *HIV* human immunodeficiency virus, *HBV* hepatitis B virus, *HCV* hepatitis C virus, *IV* intravenousγ, *BBB* blood-brain-barrier, *DDL3* delta-like ligand 3, *EGFR* epidermal growth factor receptor, *GM-CSF* granulocyte-macrophage colony-stimulating factor, *ICP* intracranial pressure, *IDH* isocitrate dehydrogenase, *IFN*-γ interferon gamma, *IL-2* interleukin-2, *MGMT* O6-methylguanine-DNA methyltransferase, *WT* wild type

Priority next steps include: (i) GBM antigen co-expression mapping to identify high-yield target combinations for TriTE development; (ii) functional evaluation of different TriTE backbone modules, including Fc-silencing, co-stimulatory, checkpoint blockade, and half-life-extending domains, in GBM; (iii) development of step-up dosing schedules and serum/CSF cytokine monitoring strategies to mitigate toxicity; (iv) comparison of BBB-penetrating delivery routes using quantitative pharmacokinetic and pharmacodynamic endpoints in orthotopic models; and (v) clinical trial designs that incorporate longitudinal antigen loss and residual disease monitoring. Together, these efforts will provide a practical path to determine whether T-cell engagers can achieve more durable disease control in GBM.

## Data Availability

Not applicable. No datasets were generated or analyzed during the preparation of this manuscript.
